# Optimizing muscle preservation during weight loss in patients with cirrhosis: A pilot study comparing continuous energy restriction to alternate‐day modified fasting for weight loss in patients with obesity and non‐alcoholic cirrhosis of the liver

**DOI:** 10.1002/osp4.70016

**Published:** 2024-10-24

**Authors:** Winston Dunn, Stephen D. Herrmann, Robert N. Montgomery, Mary Hastert, Jeffery J. Honas, Jessica Rachman, Joseph E. Donnelly, Felicia L. Steger

**Affiliations:** ^1^ Division of Gastroenterology, Hepatology and Motility, Diabetes, and Clinical Pharmacology Department of Internal Medicine University of Kansas Medical Center Kansas City Kansas USA; ^2^ Division of Physical Activity and Weight Management Department of Internal Medicine University of Kansas Medical Center Kansas City Kansas USA; ^3^ Department of Biostatistics and Data Science, Department of Internal Medicine University of Kansas Medical Center Kansas City Kansas USA; ^4^ Division of Endocrinology, Diabetes, and Clinical Pharmacology, Department of Internal Medicine Department of Dietetics and Nutrition University of Kansas Medical Center Kansas City Kansas USA

**Keywords:** body composition, cirrhosis, intermittent fasting, muscle, obesity, weight loss

## Abstract

**Introduction:**

Obesity is associated with increased morbidity in patients with advanced liver disease, but it is particularly challenging for these patients to preserve skeletal muscle mass during weight loss and accelerating sarcopenia is a concern. Alternate‐day modified fasting (ADMF) may be particularly effective for weight loss in patients with concomitant cirrhosis and obesity due to preservation of fat‐free mass (FFM).

**Methods:**

A weight loss program featuring either ADMF or a continuous low‐calorie diet (LCD) was evaluated in a 24‐week randomized clinical trial in 20 adult patients with Child‐Pugh Class A cirrhosis and obesity. Participants were randomized to either ADMF (*n* = 11) or LCD (*n* = 9). Both groups received a remotely delivered exercise program. Body composition, sarcopenia measures, and functional outcomes were assessed pre‐post.

**Results:**

Thirteen participants completed the intervention (Age = 57 ± 10; BMI = 37.7 ± 5.8 kg/m^2^). The median body weight lost in ADMF was 13.7 ± 4.8 kg (13.9% of initial body weight), while LCD lost 9.9 ± 6.9 kg (10.7% of initial body weight). Total body fat percentage decreased in both groups (ADMF: −4.1 ± 4.0%; LCD = −2.8 ± 1.4%). Fat‐free mass accounted for 34 ± 20% of total weight loss in ADMF and 38 ± 10% in LCD. Functional measures, such as timed chair stands, improved in both groups.

**Conclusion:**

This pilot study demonstrates the feasibility of the ADMF and LCD interventions to produce significant weight loss while improving body composition in patients with cirrhosis and obesity. Further research is needed to validate these findings in larger cohorts and to assess changes in muscle quality and visceral fat.

**Trial Registration:**

ClinicalTrials.gov Identifier: NCT05367596.

## Graphical abstract

Weight loss programming in patients with cirrhosis has not been well studied. In this study, we demonstrate that both a low‐calorie diet and alternate‐day modified fasting in conjunction with exercise lead to clinically relevant weight loss, better physical function, and improved cardiometabolic health in patients with concurrent obesity and cirrhosis.

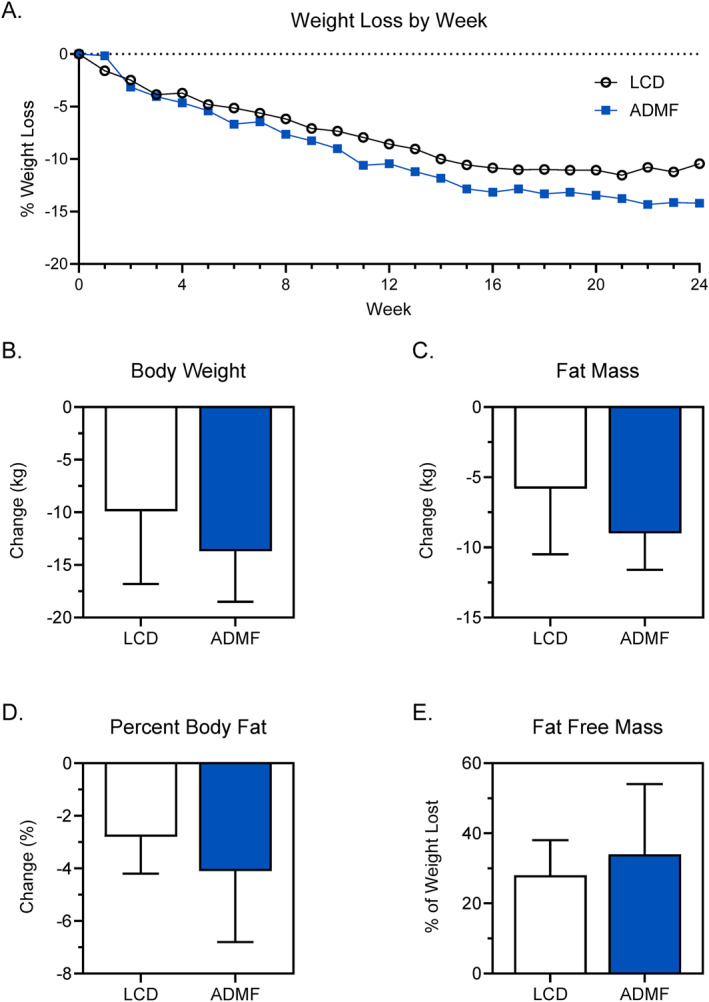

## 1 INTRODUCTION

Chronic liver disease and cirrhosis is the ninth leading cause of death in the United States^1^ and affects 4.5 million people. Just one in three people with cirrhosis know they have it, as many patients with cirrhosis remain asymptomatic until the onset of liver decompensation.^2^ Increasingly, cirrhosis is classified not a single disease entity, but one that can be subclassified into distinct clinical prognostic stages, with 1‐year mortality ranging from 1% to 57% depending on the stage.^3^ Prior to symptom onset, cirrhosis is considered to be compensated.^2^, ^4^ Complications such as ascites, variceal bleeding, hepatic encephalopathy, or nonobstructive jaundice, which can develop with cirrhosis of any origin, herald the onset of decompensated cirrhosis.^5^

About 30%–70% of those with cirrhosis have co‐existing sarcopenia, or pronounced loss of skeletal muscle mass and strength. Sarcopenia, often the result of malnutrition and low physical activity, is a predictor of adverse clinical outcomes, including survival, in this population.^6^ An American College of Sports Medicine panel found compelling evidence to support physical activity's role in modifying metabolic‐associated fatty liver disease (MAFLD) and improving clinical outcomes in patients with liver disease.^7^ However, research on patients with advanced stage liver disease is limited. A recent systematic review of 11 trials specifically intervening in patients with late‐stage liver disease supports the feasibility of exercise training in this population and found preliminary evidence for improved physical function and decreased frailty after 8–12 weeks of exercise training.^8^ Randomized‐controlled trials in patients with cirrhosis suggest that exercise programs increase exercise capacity (oxygen consumption and/or 6‐min walk duration),^9^, ^10^, ^11^, ^12^, ^13^ increase muscle mass,^9^, ^10^, ^12^, ^14^ improve muscle strength,^11^ reduce liver frailty,^14^, ^15^ reduce fatigue,^10^ and improve quality of life.^15^

Obesity is also associated with significant morbidity and mortality in those with liver disease,^16^ and obesity is an independent risk factor for decompensation of the liver and infection in those with cirrhosis.^17^, ^18^ Moreover, obesity and sarcopenia often co‐exist in patients with cirrhosis. The concurrent existence of elevated fat mass and diminished muscle mass, termed sarcopenic obesity or myopenic obesity, is associated with a poorer prognosis and increased risk of death relative to sarcopenia alone.^19^, ^20^ The benefits of interventions to improve sarcopenia and obesity are well recognized in the general population; however, most were developed for patients without cirrhosis, and their efficacy in patients with cirrhosis is less clear. Weight loss of 5%–10% in those with cirrhosis slows disease progression, but the American Association for the Study of Liver Diseases (AASLD) advises caution in recommending weight loss in this group due to the risk of exacerbating loss of fat‐free mass (FFM) which includes muscle and bone mass.^21^ Cirrhosis is associated with a state of accelerated protein breakdown characterized by a reduced respiratory quotient due to the metabolic switch from the oxidation of glucose to fatty acids and protein as primary energy sources.^22^ In this state, protein synthesis is decreased and proteolysis for gluconeogenesis is increased, and this accelerated protein degradation contributes to a greater propensity for loss of FFM and subsequent sarcopenia^23^ in this group. Further, patients with cirrhosis oxidize protein for energy after a relatively short period of fasting relative to their healthy counterparts.^24^ In healthy individuals during weight loss, approximately 15%–25% of weight loss comes from FFM,^25^, ^26^, ^27^ with better retention of FFM when exercise is prescribed.^28^

Therefore, a significant challenge in treating patients with advanced liver disease is the lack of established interventions that promote weight loss while preserving FFM, particularly skeletal muscle. Different strategies exist for either promoting weight loss or preventing sarcopenia in cirrhosis patients, but no treatments have been evaluated for achieving both goals concurrently. Approaches to improve sarcopenia in cirrhosis include increased protein intake, BCAA supplementation,^29^, ^30^, ^31^, ^32^ exercise programming,^33^, ^34^ and late‐evening snacks to delay protein degradation during the overnight fast.^32^, ^35^, ^36^, ^37^ One prior trial targeted weight loss in patients with cirrhosis, with a 16‐week intensive lifestyle intervention resulting in a loss of 5 kg of initial body weight and achieving a 5% weight loss in 52% of participants.^34^ However, changes in body composition were not assessed, and typical proportions of weight lost as FFM are unknown in this population. Previously, this research team demonstrated that losses of FFM with alternate‐day modified fasting (ADMF), a form of intermittent fasting, are <10% of total weight loss in otherwise healthy adults with overweight/obesity.^38^ Therefore, ADMF may be particularly advantageous in patients with cirrhosis where preservation of FFM is paramount.

In this trial, a 24‐week weight loss program featuring either ADMF or continuous energy restriction with a standard low‐calorie diet (LCD), which was specifically designed to minimize losses of FFM in patients with cirrhosis, was tested. Recommendations in the weight loss program to preserve FFM in both groups included adequate protein intake (1.2–1.5 g/kg/day) and an exercise program in line with AASLD guidance.^21^ To increase adherence to physical activity recommendations, remotely delivered home‐based group exercise was included, which is feasible and improves physical function, strength, and flexibility.^39^, ^40^, ^41^, ^42^, ^43^ The primary aim was to evaluate the feasibility of a 6‐month weight loss program featuring ADMF and LCD in patients with cirrhosis. Secondarily, the effects of ADMF and LCD on changes in body composition were evaluated.

## 2 METHODS

### 2.1 Participants

From April 2022 through June 2022, adults (≥18 years of age) with both non‐alcoholic Child‐Pugh Class A cirrhosis and obesity (body mass index [BMI] of 30–45 kg/m^2^) were recruited from the University of Kansas Medical Center's Hepatology Clinic. Diagnosis of cirrhosis was based on any of the following methods: (1) liver biopsy consistent with stage IV fibrosis according to the NASH Clinical Research Network scoring system; (2) liver Stiffness >14 kpa as assessed by a transient elastography exam (FibroScan® 502 Touch model; M Probe; XL Probe; Echosens); and/or (3) endoscopy or imaging findings of gastroesophageal varices.

Exclusion criteria were: (1) Child‐Pugh score >6, indicating Class B/C Cirrhosis; (2) history of ascites; (3) history of portal system encephalopathy; (4) prior history of liver cancer; (5) prior history of non‐melanoma skin cancer within the previous 5 years; (6) hepatitis C Virus without sustained virological response; (7) hepatitis B Virus without antiviral treatment; (8) diabetes (type 1 or 2) currently requiring insulin and/or sulfonylureas; (9) active alcohol consumption of >7 drinks per week in the previous 6 months; (10) disordered eating as assessed by the Eating Attitudes Test (Eat‐26) or the Binge Eating Scale; (11) inability to participate in a graduated physical activity program; (12) weight loss or gain ≥5% within the previous 6 months; (13) participation in a structured weight management program within the previous 6 months; (14) unwilling to be randomized to one or both of the treatment groups; (15) pregnancy during the previous 6 months; (16) current use of antipsychotics, untreated depression, or other major or untreated psychiatric illness which would interfere with weight management goals; (17) adherence to a specialized diet that would make it difficult or impossible to follow program guidelines; (18) lack of access to wireless Internet and/or equipment necessary for participating in a remotely delivered group education or exercise class.

Potentially eligible participants were screened for basic eligibility criteria in the clinic using an inclusion/exclusion checklist. Participants who screened positively and provided written informed consent were further assessed for health, medical and personal history. Eligible and interested participants were invited to enroll. Compensation of up to $250 was provided for study participation. The study was approved by the University of Kansas Medical Center's Institutional Review Board (STUDY00148145).

### 2.2 Intervention and randomization

This trial was a 24‐week, parallel‐arm, randomized controlled trial. Eligible participants were randomized to follow ADMF or a control diet using continuous energy restriction with a LCD. The intervention was divided into two phases: a 12‐week weight loss phase and a 12‐week weight loss maintenance phase. Study data were collected and managed using REDCap electronic data capture tools hosted at the University of Kansas Medical Center.^44^, ^45^

*Intervention diets*. During the weight loss phase, ADMF participants followed a very‐low energy diet (600–850 kcal/day) 2 or 3 days per week and were asked to follow general healthy eating guidance as outlined by ChooseMyPlate.gov on opposite days. The very‐low energy diet prescription consisted of four protein supplements (three shakes and one meal bar), one portion‐controlled entrée, and 2–3 cups of vegetables. In contrast, LCD participants followed a low energy diet (1200–1600 kcal/day) each day using four portion‐controlled shakes, two portion‐controlled entrees, and at least five total servings of fruits and vegetables.

All participants were provided with identical instructions for choosing protein supplements (≥15 g protein) and portion‐controlled meals (≤350 kcal, ≥15 g protein, ≤3 g saturated fat, ≤10 g sugar). Additionally, meal replacement shakes (Orgain, Inc.© Grass‐Fed Whey Protein Powder; 21 g protein/serving) and protein bars (ProfilePlan, LLC, 15 g protein/serving) were provided to participants. Participants were educated on spacing protein supplements and entrees throughout the day, and both groups were instructed to have their last shake within 2 h of going to sleep to minimize protein catabolism during the overnight fast. Recommendations for general healthy eating for weight loss were delivered through a comprehensive weight management program.

During the 12‐week weight loss maintenance phase, participants were advised to continue following the diet at a level that preserved weight loss. ADMF participants were advised to incorporate 1–2 days of a very‐low energy diet per week as needed to maintain body weight. LCD participants were counseled to modify the energy restriction to maintain body weight.

*Exercise*. Remotely delivered group exercise classes were provided to both groups twice per week via Zoom video conferencing software (Zoom Inc.). Sessions consisted of a warm‐up (∼10 min), Moderate Physical Activity (MPA 3–6 metabolic equivalents; ∼30 min), and a cool down (∼5 min). A combination of body weight and resistance band exercises was used during each session and the exercises progressed in intensity and resistance over time. Intensity of physical activity was self‐monitored using Fitbit Versa 2 (Fitbit LLC) monitors provided by the study. At least 150 min of moderate–vigorous physical activity (MVPA) was recommended and this was achieved with a combination of the group classes and independent exercise. Exercise goals were ramped up from 60 min of MVPA per week in Week 1 to ≥150 min MVPA in Week 4; the exercise goal remained at ≥150 min of MVPA per week through the end of the intervention. Quality MVPA minutes were calculated using the sum of moderate‐intensity physical activity and 2 × minutes of vigorous‐intensity activity. Weekly steps were recorded using Fitbit Versa 2 activity monitors (Fitbit, Inc.) and both steps and weekly minutes of MVPA were self‐reported via a REDCap survey.

*Group* *support* *and self‐monitoring*. A comprehensive weight management program was employed via weekly group meetings led by a registered dietitian. Weekly group classes lasted approximately 1 h and consisted of group discussion, a nutrition, exercise, or lifestyle behavior lesson, followed by weekly goal setting and homework assignments. REDCap surveys were used to collect self‐report data specific to the intervention groups each week. In this weekly survey, participants reported the weekly total number of protein supplements, portion‐controlled entrees, fruit, vegetables, compliance to protein spacing, steps, quality exercise minutes (MVPA), and barriers to compliance. Aggregate weekly data were reviewed in the group weight management sessions. Additionally, individual feedback was provided via e‐mail or phone when additional support was needed.

### 2.3 Anthropometry and body composition

Height was collected using a stadiometer and weight was assessed to the nearest 0.1 kg with a calibrated scale (Model #PS6600, Belfour). Height and weight measurements were assessed in duplicate, and the average was used to calculate body mass index (BMI; kg/m^2^). Dual energy X‐ray absorptiometry (DXA) at the KUMC Division of Physical Activity and Weight Management (Prodigy Advance Plus, GE) was used to assess body composition. Fat loss was assessed as an absolute change from baseline. For all participants who lost at least 3% of their initial body weight, change in FFM was assessed both in absolute change from baseline and as percent weight loss from FFM ([Δ fat‐free mass/Δ weight loss] × 100). Leg muscle mass was estimated from DXA using the prediction equation by McCarthy et al. (0.78 × leg lean–1.07) as a proxy for change in muscle mass.^46^

### 2.4 Liver health and functional assessments

A transient elastography exam (FibroScan® 502 Touch model; M Probe; XL Probe; Echosens) was conducted to assess liver stiffness at baseline to determine study eligibility. The ability to carry out normal activities of daily living was determined via the Karnofsky Performance Status^47^ pre‐ and post‐intervention. The Liver Frailty Index (LFI) was assessed using grip strength, timed chair stands, and balance testing pre‐ and post‐intervention.^48^, ^49^ Grip strength was measured in kilograms using a handheld dynamometer in the dominant hand; the average of three trials was calculated for analysis. Timed chair stands were measured as the number of seconds required to complete five chair sit‐to‐stands with arms folded across the chest. Balance testing was measured as the number of seconds that each participant could balance in three positions (feet placed side‐to‐side, semi‐tandem, and tandem) for a maximum of 10 s each. LFI was calculated using the following equation:

LFI=(–0.330×sex−adjustedgripstrength)+(–2.529×numberofchairstandspersecond)+(–0.040×balancetime)+6.

The classifications of frailty were determined using previously established cutoffs of the LFI with “robust” defined as LFI <3.2, “prefrail” defined as LFI between 3.2 and 4.4, and “frail” defined as LFI ≥4.5.^48^, ^49^

### 2.5 Laboratory assessments

A complete blood count, comprehensive metabolic panel, prothrombin time, international normalized blood ratio test, and a lipid panel were collected and processed via the University of Kansas Hospital laboratory. Laboratory assessments after a 12‐h fast were used to screen for Child‐Pugh B/C cirrhosis at baseline and were collected again at weeks 12 and 26 of the intervention.

### 2.6 End of study survey

All participants who received the intervention were sent a survey via REDCap at the end of their participation to provide feedback on the intervention as a whole and for intervention components. All question responses were in the form of a Likert 1–5 rating scale.

### 2.7 Statistical analysis

Descriptive statistics were used to summarize the results by group. Counts and percentages were used for categorical data. Given the small sample sizes, the median and median absolute deviation (MAD) were used as measures of central tendency and variability for continuous variables and are presented as median ± MAD. The raw MAD was adjusted by a factor of 1.4826 to ensure that if the underlying population is normally distributed, the MAD would be a consistent estimator of the population standard deviation. Changes in weight and BMI were assessed using Wilcoxon signed‐rank tests.

## 3 RESULTS

### 3.1 Participant characteristics and retention

**FIGURE 1:**
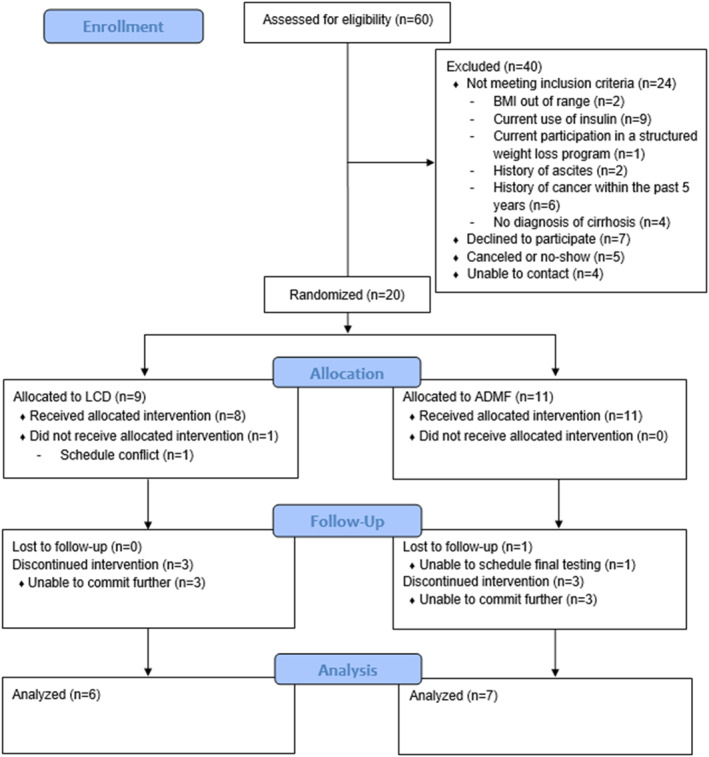
CONSORT diagram.

Sixty individuals were screened and 20 were randomized to either the continuous low‐calorie diet (LCD; *n* = 9) or alternate‐day modified fasting (ADMF; *n* = 11; Figure 1). Participants were excluded due to not meeting inclusion criteria (*n* = 24), failure to complete baseline testing (*n* = 4), inability to contact (*n* = 4), or because they declined to participate (*n* = 7). Of the 20 participants randomized, 19 received the intervention (LCD: 8, ADMF: 11) and 1 participant allocated to LCD withdrew prior to the intervention starting. Three participants from each group withdrew from the intervention and one additional participant from ADMF was unable to schedule the final testing visit. Therefore, 13 individuals completed the intervention, provided final data, and were included in the analysis (LCD: 6; ADMF: 7). Retention was 67% for LCD and 64% for ADMF.

Study completers were age 57 ± 10 years, primarily female (61%), and primarily white (77%), with a BMI of 37.7 ± 5.8 kg/m^2^ (Table 1). Fifty‐four percent of participants had type 2 diabetes mellitus. No serious adverse events or liver decompensation were reported during the 24‐week intervention.

**TABLE 1:** Participant demographics and baseline characteristics.

Characteristic	All participants (*n* = 13)	LCD (*n* = 6)	ADMF (*n* = 7)
Demographics			
Age, median (MAD), y	57 (10)	55 (8)	57 (13)
Sex, no. (%)			
Female	8 (61)	4 (67)	4 (57)
Male	5 (39)	2 (33)	3 (43)
Race, no. (%)			
American Indian/Alaska native	2 (15)	1 (17)	1 (14)
Black	1 (8)	0 (0)	1 (14)
White	10 (77)	5 (83)	5 (71)
Ethnicity, no. (%)			
Hispanic or Latino	0 (0)	0 (0)	0 (0)
Not Hispanic or Latino	12 (92)	5 (83)	7 (100)
Unknown or not reported	1 (8)	1 (17)	0 (0)
Body mass index (kg/m^2^)	37.7 (5.8)	40.0 (8.2)	33.9 (13.3)
KPSS, *n* (%)			
1	6 (46)	3 (50)	3 (43)
2	6 (46)	2 (33)	4 (57)
3	1 (8)	1 (17)	0 (0)
Type 2 diabetes mellitus, *n* (%)	7 (54)	4 (67)	3 (43)

### 3.2 Attendance and adherence to program recommendations

**FIGURE 2:**
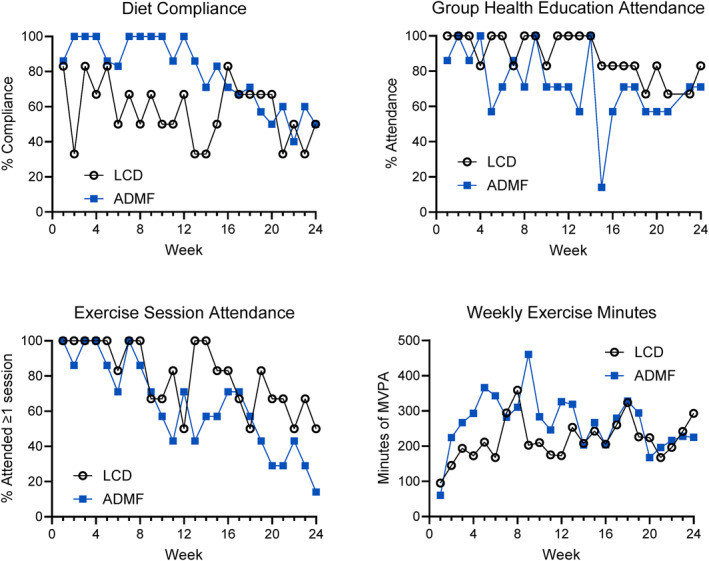
Compliance to lifestyle program components and attendance. (A) Percentage of days compliant (yes/no) to the prescribed diet guidelines each week during the intervention. (B) Percentage of each group who attended weekly group health education meetings. (C) Percentage of each group who attended at least one remotely delivered exercise session. (D) Median weekly minutes of moderate–vigorous physical activity from combined group exercise classes and independent physical activity.

Compliance to intervention recommendations and attendance is shown in Figure 2. Attendance at weekly group meetings was 89% and 72% for LCD and ADMF, respectively. The percentage of participants who attended at least one exercise session each week was 83% for LCD and 66% for ADMF. The average total quality minutes per week of moderate–vigorous activity reported was 254 ± 216 for LCD and 381 ± 399 for ADMF. Average weekly steps were 44,192 ± 39,171 for LCD and 53,213 ± 29,019 for ADMF.

The LCD group consumed 10 ± 5 portion‐controlled entrees, 21 ± 10 protein shakes, 15 ± 4 cups of fruit, and 15 ± 7 cups of vegetables weekly. The ADMF group consumed 4 ± 3 portion‐controlled entrees, 21 ± 4 protein shakes, 8 ± 4 cups of fruit, and 20 ± 4 cups of vegetables weekly. On average, both groups reported spacing protein intake to every 3–4 waking hours on 5 days per week on average. ADMF participants followed a very‐low calorie diet on 3 ± 1.5 days per week during the weight loss phase, and 2 ± 1.5 days during the weight maintenance phase.

### 3.3 Weight and body composition

**FIGURE 3:**
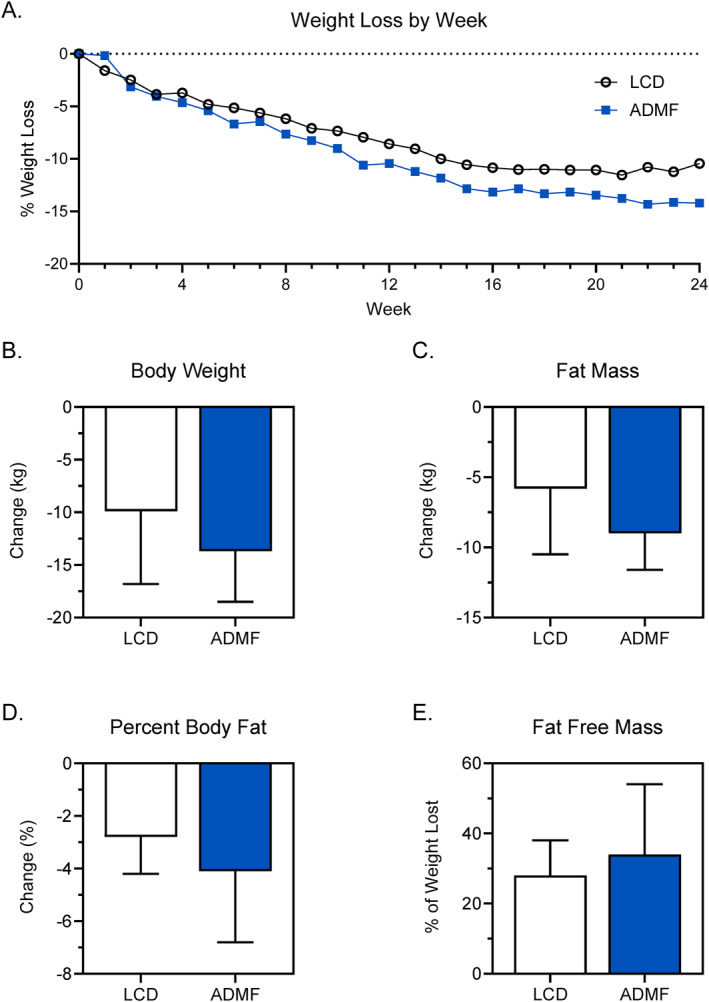
Weight and body composition changes after a 24‐week intervention featuring a continuous LCD or ADMF using a very‐low calorie diet 2–3 days weekly. (A) Weekly relative (%) weight loss. Weights were self‐reported for all time points except for baseline (week 0) and weeks 24. (B) Change in body weight after 24 weeks using metabolic weight. Change in fat mass (C), percent body fat (D), and the percentage of weight lost as fat‐free mass (FFM) (E) measured by DXA. ADMF, alternate‐day modified fasting; DXA, dual X‐ray absorptiometry; LCD, low calorie diet.

Both LCD (−9.9 ± 6.9 kg; *p* = 0.03) and ADMF (−13.7 ± 4.8 kg; *p* = 0.02) produced clinically relevant weight loss (Table 2; Figure 3A,B). BMI decreased by 3.8 ± 3.0 kg/m^2^ (*p* = 0.03) for LCD and 4.7 ± 2.7 kg/m^2^ (*p* = 0.02) for ADMF. LCD decreased body fat percentage by 2.8 ± 1.4%, whereas ADMF decreased body fat percentage by 4.1 ± 2.7% (Figure 3D). All completers achieved at least 3% weight loss and were therefore included in the percent weight loss from FFM calculation. The percent weight lost from FFM was 28 ± 10% for LCD and 34 ± 20% for ADMF. One participant in each group limited FFM loss to our pre‐specified goal of less than 20% of total weight loss. Change in leg lean mass, a proxy for muscle mass, was −1 ± 0.3 kg for LCD and −0.8 ± 1 kg for ADMF.

**TABLE 2:** Changes in weight, body composition, cardiometabolic risk factors, laboratory measures, liver health, and physical function outcomes after a 24‐week weight management intervention for patients with cirrhosis and obesity featuring either a LCD or ADMF.

Outcome	Baseline		Follow‐up		Change (pre—Post)	
	LCD	ADMF	LCD	ADMF	LCD	ADMF
Weight loss, kg	108.2 (25.9)	99.9 (16.5)	96.0 (23.7)	90.8 (12.9)	−9.9 (6.9)^a^	−13.7 (4.8)^a^
Weight loss, %					−10.7 (6.9)	−13.9 (1.1)
Body mass index (kg/m^2^)	40.0 (2.9)	33.9 (3.4)	36.2 (4.4)	29.6 (1.6)	−3.8 (3.0)^a^	−4.7 (2.7)^a^
Body fat, %	47.3 (3.5)	43.3 (5.6)	45.8 (3.1)	38.6 (8.3)	−2.8 (1.4)	−4.1 (4.0)
Fat–Mass, kg	51.0 (11.3)	42.3 (7.2)	42.8 (14.8)	35.3 (8.0)	−5.8 (4.7)	−9.0 (2.6)
Fat‐free mass, kg	59.9 (6.3)	56.4 (9.7)	56.0 (9.0)	49.5 (11.5)	−5.4 (2.4)	−3.7 (1.5)
Leg lean muscle, kg	11.8 (1.4)	12.5 (1.0)	10.8 (1.6)	11.4 (2.6)	−1 (0.3)	−0.8 (1)
Systolic BP, mm Hg	130 (20)	127 (5)	126 (15)	126 (6)	−3 (14)	−4 (9)
Diastolic BP, mm Hg	74 (2)	80 (9)	70 (10)	86 (12)	2 (13)	3 (13)
Heart rate, beats/min	74 (5)	65 (9)	60 (5)	68 (12)	−13 (10)	1 (6)
Hemoglobin, g/dL	14.7 (0.2)	14.8 (0.6)	14.6 (0.5)	15.2 (0.3)	0.2 (0.7)	0.4 (0.4)
Hematocrit, g/dL	43.0 (1.1)	44.3 (2.4)	43.4 (3.3)	44.7 (1.3)	1.7 (2.7)	0.4 (0.9)
Platelet count, k/uL	188 (36.3)	187 (59.3)	171 (93.4)	189 (56.3)	−12 (17)	12 (11)
BUN, mg/dL	11.5 (3.7)	11 (3.0)	15 (3.7)	15.0 (3.0)	1.5 (3.7)	5 (1.5)
Creatinine, mg/dL	0.9 (0.2)	0.8 (0.2)	0.8 (0.2)	0.9 (0.1)	−0.05 (0.07)	0.04 (0.04)
Albumin, g/dL	4.2 (0.4)	4.3 (0)	43 (0.4)	4.4 (0.3)	−0.05 (0.2)	0 (0.2)
AST, u/L	37 (22.2)	19 (4.5)	34 (22.2)	19.0 (3.0)	−2.0 (10.4)	−4 (10.4)
ALT, u/L	32.5 (14.1)	26.0 (14.8)	36.0 (31.1)	18.0 (3.0)	2.0 (14.1)	−14.0 (14.8)
Alkaline phosphatase, u/L	85.0 (46.0)	88.0 (13.3)	85.0 (43.0)	79.0 (31.1)	−1.0 (4.5)	−8.0 (14.8)
Glucose mg/dL	98 (16)	98 (18)	97 (13)	90 (7.4)	−6 (14)	−13 (25)
Total cholesterol, mg/dL	166 (37)	176 (33)	152 (23)	161 (13)	−5 (39)	−28 (18)
LDL cholesterol, mg/dL	102 (11)	116 (16)	83 (33)	100 (16.3)	−2 (20)	−27 (16)
HDL cholesterol, mg/dL	52 (16)	49 (12)	52 (10)	51 (9)	−1 (5)	3 (5)
Triglycerides, mg/dL	143 (24)	173 (22)	91 (9)	114 (22)	−17 (72)	−31 (39)
Timed Chair Stands, s	14.8 (4.2)	15.3 (5.8)	9.7 (2.1)	7.9 (2.1)	−4.9 (3.0)	−5.0 (2.7)
Grip strength, kg	7.5 (4.7)	14.0 (10.9)	8.2 (4.0)	10.0 (9.4)	2.3 (3.2)	−1.3 (3.5)
Liver Frailty Index	2.0 (0)	2.0 (0)	2.0 (0)	2.0 (0)	0 (0)	0 (0)
KPSS score	95 (7.4)	90.0 (0)	90.0 (7.4)	100 (0)	0 (14.8)	0 (14.8)

*Note*: Data presented are median and median absolute deviation (MAD) values. For KPSS follow (ADMF) 80–100. For KpSS change −10 to 10. For kpss change (LCD) −30 to 10.

Abbreviations: ADMF, alternate‐day modified fasting; ALT, alanine transaminase; AST, aspartate aminotransferase; BP, blood pressure; BUN, blood urea nitrogen; HDL Cholesterol, high‐density lipoprotein cholesterol; KPSS, Karnofsky Performance Status Scale; LCD, daily low‐calorie diet; LDL Cholesterol, low‐density lipoprotein cholesterol.

^a^ MAD for albumin at baseline in ADMF group is 0 because more than half of the observed values were equal, and the range of values was 3.9, 4.8.

*Within‐group changes significant at *p* < 0.05.

### 3.4 Liver health, physical function, and cardiometabolic risk factors

**FIGURE 4:**
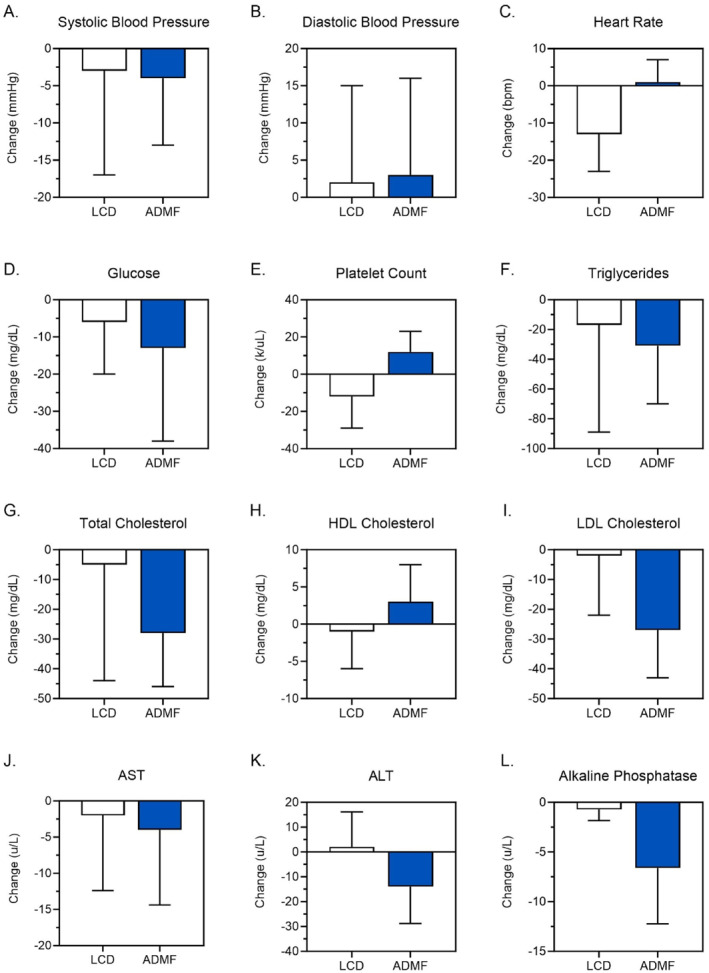
Markers of cardiometabolic health and liver function. Changes in (A) systolic blood pressure, (B) diastolic blood pressure, (C) heart rate, (D) glucose, (E) platelet count, (F) triglycerides, (G) total cholesterol, (H) high‐density lipoprotein cholesterol, (I) low‐density lipoprotein cholesterol, (J) aspartate aminotransferase, (K) alanine transaminase, and (L) alkaline phosphatase after 24 weeks.

Cardiometabolic and laboratory changes are presented in Table 2 and displayed in Figure 4. Neither LCD nor ADMF significantly changed systolic (LCD: −3 ± 14 mmHg; AMDF: −4 ± 9 mmHg) or diastolic (LCD: 2 ± 13 mmHg; AMDF: 3 ± 13 mmHg) blood pressure. Fasting glucose was also not impacted by either intervention (−6 ± 14 mg/dL in LCD and −13 ± 25 mg/dL in ADMF). Triglycerides were reduced by 31 ± 39 mg/dL in ADMF but were relatively unchanged in LCD 17 ± 72 mg/dL in LCD. HDL cholesterol did not change in either LCD (−1 ± 5 mg/dL) or ADMF (3 ± 5 mg/dL). Similarly, LDL cholesterol did not change in either LCD (−2 ± 20 mg/dL) or ADMF (−27 ± 16 mg/dL). ADMF significantly decreased alanine transaminase (ALT) by 14.0 ± 14.8 U/L but did not have a significant effect on AST (−4 ± 10.4 U/L) or alkaline phosphatase (−8.0 ± 14.8 IU/L). LCD did not significantly affect ALT (2.0 ± 14.1 U/L) AST = −2.0 ± 10.4 U/L or alkaline phosphatase (‐1.0 ± 4.5 IU/L). The time it took to complete the five chair stands test improved in both groups (LCD: −4.9 s, *p* = 0.03; ADMF: −5.0 s, *p* = 0.02).

### 3.5 Diet difficulty, hunger, quality of life, and participant satisfaction

Thirteen participants (LCD: 6, ADMF: 7) responded to the exit survey. All ADMF participants were satisfied with the diet. Most (4/5) LCD participants were satisfied with their current diet. Participants were asked how difficult the dietary component of the intervention was: 2/7 ADMF and 2/6 LCD participants responded “not at all difficult,” 4/7 ADMF and 3/6 LCD responded “slightly difficult,” while 1/7 ADMF and 1/6 LCD responded “moderately difficult.” Hunger was not a factor in diet difficulty; one participant from each group was neutral regarding hunger as a significant barrier, while all other participants disagreed that hunger was problematic. Notably, all participants from both groups said they were likely to continue using the diet recommendations.

When asked whether the physical activity recommendations were achievable and sustainable, most (6/7) ADMF participants agreed with this statement and one participant was neutral. Most (5/6) LCD participants either agreed (4/6) or strongly agreed (1/6) that the physical activity recommendations were achievable and sustainable, while one participant disagreed. When asked whether the remote exercise classes decreased barriers for achieving the exercise goal, responses were mixed: in LCD, 4/6 participants answered “yes” or “definitely yes,” while one participant was uncertain and one answered “no;” in ADMF, 4/7 answered yes, 2/7 were uncertain, and 1/7 answered “no.” Responses were also mixed when asked how likely they were to continue following the intervention exercise recommendations: 1/7 ADMF and 2/6 LCD said they were “not at all likely” to continue, 4/7 ADMF said they were “somewhat likely,” 2/6 LCD and 1/7 ADMF said they were “probably likely,” and 2/6 LCD and 1/7 ADMF said they were “certainly likely” to continue the exercise program.

All LCD participants agreed that the intervention improved their quality of life. In ADMF, most (6/7) participants also agreed with experiencing quality of life improvements. Regarding program design, 4 of the 13 total participants thought that the total meeting frequency (education + exercise sessions) was too high, while most (9/13) thought there were just the right number of meetings. Lastly, all participants either agreed or strongly agreed that the intervention reduced their risk of chronic diseases.

## 4 DISCUSSION

This study primarily aimed to evaluate the practicality and safety of a tailored weight loss program for individuals with both cirrhosis and obesity. It achieved notable success in participant retention and engagement, with roughly two‐thirds of participants completing the program and high attendance in both dietary and exercise components. This study demonstrated high adherence to program recommendations with minimal adverse effects, indicating the potential for such tailored interventions in this patient population. Participant satisfaction was generally high, and the majority found the program's dietary and exercise recommendations both feasible and sustainable, with a strong inclination to continue these practices post‐study. Notably, hunger was not a significant barrier to adherence in either group.

Both LCD and ADMF significantly reduced body weight, body mass index, and body fat after 24 weeks. Approximately 28%–34% of the weight lost was attributed to FFM based on the relative proportion of fat loss to FFM change. The primary aim of this intervention was to optimize fat loss while preserving FFM. To address this goal, several recommendations, including protein intake of at least 1.2 g/kg/day,^21^ spacing protein consumption approximately every 4–5 h throughout the day to stimulate muscle protein synthesis multiple times,^50^, ^51^ and promoting a mixed carbohydrate and protein snack within 2 hours before bed to delay protein catabolism overnight^36^ were implemented.

Weight loss trials in patients with cirrhosis are limited, with only one prior trial targeting weight loss in this population that did not report changes in body composition.^52^ Therefore, the typical ratio of FFM to fat mass loss in this group remains unknown. Our data indicate that patients with cirrhosis lose a higher proportion of weight from FFM compared with healthy subjects, even when multiple aspects of the intervention are designed to spare lean mass. Notably, despite a higher anticipated loss of FFM, changes in leg lean mass, serving as a proxy for changes in skeletal muscle, indicate that only 6%–8% of skeletal muscle was lost during the intervention. Therefore, changes in hydration and water retention after calorie restriction and intermittent fasting may underly changes in percent weight lost from FFM. It is plausible that patients with cirrhosis may require a protein intake greater than 1.2 g/kg/day to mitigate stored protein catabolism, although the impact of higher protein intake on palatability, diet satisfaction, and compliance to dietary recommendations remains uncertain. Future trials should investigate typical body composition changes during weight loss in patients with cirrhosis to establish a range of normal in this group. Moreover, intermittent energy restriction and other forms of intermittent fasting should be investigated in subsequent clinical trials with more precise measures of skeletal muscle and liver health (e.g., magnetic resonance imaging) to determine whether the risk of elevated protein catabolism with very‐low energy intake and/or an extended overnight fast increases the risk of sarcopenia in patients with cirrhosis.

Despite a loss of muscle mass, the weight loss and exercise program improved measures of physical function. Both groups required less time to complete the timed chair stand test, although no change was observed in the liver frailty index or liver enzymes. Thus, while losses of FFM exceeded our goal of 20% of total weight loss, concomitant declines in liver health or muscle function were not observed. Our results are in line with improvements in the Timed Up and Go test observed by Rooman et al.^9^ Muscular strength and function are not linearly related to muscle mass, and while muscle strength and muscle mass are correlated, muscle strength may be more closely related to loss of physical function.^53^ Additional research on muscle quality and function should be conducted, as it is possible that muscle mass and physical function should be concurrently assessed in this population. Though the self‐reported minutes of MVPA exceeded program recommendations, ∼17% of LCD and ∼33% of ADMF attended fewer than one group resistance exercise class each week, on average. Follow‐up studies using either supervised exercise and/or measures of muscular quality and strength should be considered when evaluating the impacts of weight loss on muscle health in this group. Lastly, anti‐obesity medications are increasingly used in weight management programming but have not been tested in patients with cirrhosis.^54^ One trial assessed the impact of liraglutide in patients with MAFLD and noted greater resolution of MAFLD and reduced progression of fibrosis relative to controls.^55^ Reductions in FFM are a concern with anti‐obesity medications, and therefore, these medications may be contraindicated in patients with cirrhosis and should be further explored in this group.^56^

In conclusion, this study demonstrated that energy restriction using either a LCD or intermittent energy restriction is feasible and results in clinically meaningful weight loss in patients with cirrhosis with no serious adverse events. Future interventions utilizing both dietary methods are needed to replicate these results and further explore the effects of both regimens on changes in muscle quantity, muscle quality, physical function, and liver health. Increasing protein intake beyond the recommended 1.2 g/kg/day during weight loss may be required to reduce protein catabolism. Lastly, efficacy trials are needed to assess the ability of structured exercise programs to mitigate losses of FFM during weight loss in patients with cirrhosis.

This study has notable strengths but also limitations. To our knowledge, this is the first trial to assess the impact of weight loss on body composition in patients with both cirrhosis and obesity. Additionally, it stands out as the longest clinical trial to date involving any lifestyle intervention within this population. Notably, the study employed robust measures of body composition using DXA and illustrated that significant weight changes and improvements in liver health and physical function are achievable within this population. However, limitations include a small sample size and insufficient statistical power to detect between‐group differences due to the pilot nature of the trial. Furthermore, this study lacked in‐depth assessments of muscular strength and objective measures of moderate‐to‐vigorous physical activity.

## AUTHOR CONTRIBUTIONS

Conceptualization: Felicia L. Steger, Winston Dunn. Joseph E. Donnelly. Methodology: Felicia L. Steger, Winston Dunn, Joseph E. Donnelly, Stephen D. Herrmann, Jeffery J. Honas. Study Execution: Felicia L. Steger; Mary Hastert, Stephen D. Herrmann, Winston Dunn, Jessica Rachman. Statistical Analysis: Robert N. Montgomery. Writing—Original Draft: Felicia L. Steger and Winston Dunn. Writing—Review and Editing: all authors.

## CONFLICT OF INTEREST STATEMENT

The authors declare no conflicts of interest.
